# Literature Review of Anatomical Variations: Clinical Significance, Identification Approach, and Teaching Strategies

**DOI:** 10.7759/cureus.14451

**Published:** 2021-04-13

**Authors:** Abdulrahman Alraddadi

**Affiliations:** 1 Basic Medical Sciences, Anatomy, King Saud bin Abdulaziz University for Health Sciences (KSAU-HS), Riyadh, SAU; 2 Basic Medical Sciences, Anatomy, King Abdullah International Medical Research Center (KAIMRC), Riyadh, SAU

**Keywords:** anatomical variations, anatomical education, anomalies, cadaveric dissection, medical education

## Abstract

This article is a comprehensive literature review on anatomical variations, shedding some light on their clinical significance, identification approaches, and teaching strategies. Anatomical variation is a normal presentation of body structure with morphological features different from those that have been classically described in several anatomy textbooks. Under normal circumstances, it has no impact on the function of the structure. However, it may influence different aspects of clinical practice. As a result, accurate knowledge of common anatomical variations in the treated area may improve clinical practice outcomes. On the other hand, anatomical variations are usually identified during routine dissection and clinical practice, including preoperative imaging and surgical procedures. Additionally, scientific research, such as observational studies using cadaveric dissection, medical images, and evidence-based anatomy, are effective approaches to identify anatomical variations. With regard to the teaching of anatomical variations, cadaveric dissection is the most commonly used and recommended learning tool for teaching anatomy and relevant variations in medical schools. However, the literature emphasizes introducing anatomical variations in the clinical phase of medical curricula and postgraduate training of surgical and radiological programs. The current study suggests creating a registry of anatomical variations encountered during practice that may enhance best care and prevent any confusion about those variations. In addition, there is a need to conduct more educational studies to research the best learning strategies for teaching and assessing anatomical variations in the medical curricula.

## Introduction and background

Choosing which running shoes to buy is evidence that our feet are not all the same. Therefore, normal does not always mean the same, especially regarding our bodies. Anatomical variation is a normal presentation of body structure with morphological features different from those described in the literature. It involves many types and forms of variations, including muscle structure, attachments, and innervation, ligament attachments and morphology, blood vessel branching, nerve patterns and positions, and bone morphology and accessory bones [1-7]. It has been found that some of these anatomical variations are closely related to ethnic groups [8].

Under normal circumstances, anatomical variations do not affect the function of the organ. However, clinically, anatomical variations play a significant role in diagnosis and therapeutic procedures in which they may require more attention or specific arrangements [9]. Anatomical variations can be closely linked to race, environmental adaptation, geographical region, and exposure to chemicals or radiation. Thus, identifying these variations will reflect positively on the clinical outcomes by minimizing any confusion or malpractice.

Identification of anatomical variations usually occurs during routine dissection and imaging investigations or surgical procedures. A study found that experienced clinicians reported seeing variations in their practice on a monthly (39%), weekly (25%), and daily (21%) basis [10]. It has been reported that ignorance of these variations was responsible for 10% of malpractice cases [11]. Therefore, the current review article will discuss the anatomical variations, focusing on their significance in clinical practice, approaches to identifying them, and how to teach them in medical schools’ curricula.

## Review

Significance of anatomic variations in clinical practice

The influence of anatomical variations on different aspects of clinical practice can be categorized into the predisposition to illness, symptomatology, clinical examinations, and treatment of patients, including surgery [12]. Previous studies found that individuals with left coronary artery dominance have a higher risk of in-hospital mortality and myocardial reinfarction than those with right coronary artery dominance [13]. The anatomy of the vermiform appendix also showed variable positions, including pelvic (56%), subcecal (19%), retroileal (13%), retrocecal (7%), ectopic (4%), and pre-ileal (2%) [14]. These variations may mislead the diagnosis in patients with acute appendicitis and may result in severe complications, such as perforation and sepsis.

A large body habitus can produce low-quality images in clinical examinations, affecting the accuracy of clinical interpretations of the images [15]. Another rare anomaly that can be seen during the clinical investigation of the chest is the azygos lobe of the lung [16,17]. It was seen in 0.4% of chest radiograms and may be confused clinically with a bulla or abscess. The routine clinical practice of measuring blood pressure may also interfere with the higher division of the brachial artery, which can occur in 20% of cases [18,19]. Arterial variation is common in the upper extremities. Therefore, accurate knowledge of these variations may help prevent limb complications such as injury, thrombosis, gangrene, or even amputation of limbs [20].

Furthermore, a safe laparoscopic cholecystectomy requires accurate knowledge of the cystic artery anatomy variations [21]. Originally, the cystic artery is a branch of the right hepatic artery, but it may also arise from other arteries, including the left hepatic artery, the hepatic artery proper, the common hepatic artery, the superior pancreaticoduodenal artery, the superior mesenteric arteries, or even the gastroduodenal artery [22].

The pectoralis minor also shows variable anatomy that is worth considering in clinical practice. The pectoralis minor is a significant anatomical landmark on the chest wall providing access to many related structures in the clavipectoral and axial regions [23]. However, the muscle shows several variations, including the origin and insertion of the attachment sites [24, 25], muscle length [26], vasculature [27], and innervation [28]. Hence, knowing the anatomical details of the pectoralis minor is essential for many clinical applications and surgical procedures such as pectoralis minor release and nerve block [29, 30].

In an attempt to increase awareness of anatomical variations in general surgery, Kowalczyk and Majewski discussed the variable anatomy that may influence the outcomes of common surgical procedures [31]. The listed surgeries included cholecystectomy, inguinal hernia repair, hepatobiliary surgery, spleen, colon, thyroid, breast, axillary, pancreatic, and gastric surgery. They recommended closing this gap, usually cited as a technical error in surgical injuries, through introducing dissection courses and using preoperative imaging techniques. Therefore, accurate knowledge of the anatomical variations may improve the clinical practice outcomes.

Approaches to identifying anatomical variations

Practitioners and anatomical scientists play a significant role in identifying anatomical variations. The typical traditional approach to identifying anatomical variations was through cadaveric dissection [12]. In contrast, some of the variations were identified in clinical practice during physical examinations, imaging investigations, and surgical procedures. In addition, the literature showed that scientific research, including observational studies and evidence-based anatomy, boosted identifying any anatomical variations.

The literature review showed several studies that reported rare cases of anatomical variations identified during routine laboratory dissections or surgical procedures. Benes and Kachlik [32] reported two cases of variable branching of the musculocutaneous and median nerves found during a routine dissection course. Kim et al. [33] also presented a novel variation identified during routine dissection in which the posterior division of the femoral nerve was penetrated by the ascending branch of the lateral circumflex femoral artery. Preoperative medical scans helped Vigneshwaran and his colleagues [34] to identify double variations of a horseshoe kidney and an accessory renal artery in a challenging case of low rectal cancer planned for laparoscopic resection. In addition, during preoperative diagnosis of a living liver donor, Gündoğdu and Kebapçı [35] observed an accessory right hepatic artery originating from the dorsal pancreatic artery and a middle hepatic artery arising from the pancreaticoduodenal artery. These preoperative observations of anatomical variations guided the surgeons to safe surgical resection of the cancer, and they searched for a new liver donor [34,35].

However, more significant results of anatomical variations are commonly reported through observational studies that inspect larger numbers of cadavers and medical images and outline their implications in health practice. A recent study assessed the challenges in using the posterior inferior cerebellar artery to the anterior inferior cerebellar artery bypass in 10 cadavers [36]. The anatomy of the two arteries was evaluated. Both arteries showed morphological and parametric variations, which made the bypass impossible. Another study examined the morphological anatomy of the sphenoid sinus using computerized tomography images of 320 adult patients [37]. The results revealed various pneumatization types, variable lateral extensions, multiple inter-sphenoid septa, and protrusion of the internal carotid artery into the sphenoid sinus. Identifying such variations is essential prior to any skull base surgery using the endoscopic endonasal transsphenoidal approach.

To enhance the findings of reported anatomical variations, both Yammine [38] and Henery et al. [39] introduced a new concept in the field: evidence-based anatomy. Evidence-based anatomy applies systematic review and meta-analysis principles to appraise and synthesize previous anatomical findings to generate a large, pooled sample size that is more likely to be accurate and reflect true population statistics and associations. Asghar et al. [25] published a systematic review and meta-analysis study about the prevalence of anomalous insertion of the pectoralis minor that included 4,146 shoulders pooled from 25 studies. Taterra et al. [40] also discussed the prevalence and anatomy of parathyroid glands in a meta-analysis study that included 7,005 patients and 23,519 parathyroid glands from 26 studies.

Nevertheless, a high clinical skill level is required to recognize and deal with these variations in clinical practice [12]. Thus, the current paper suggests creating a health informatics database to collect the common anatomical variations experienced in clinical practice. With the aid of information technology, the surgery and medical imaging units should gather any observed anatomical variations presented during clinical encounters. Those data should be compiled into a registry that includes the description of variations, demographic data, and the anticipated effects on routine clinical practice. Creating such a database will increase awareness of these variations, specifically to new resident doctors or medical interns that have joined the healthcare organization. The database of anatomical variations will support national health records by identifying common anatomical variations among ethnic groups. For example, the supernumerary heads of the biceps brachii muscle were reported commonly in South Indians [8]. Therefore, the registry can provide a benchmark and pattern identification and an opportunity to assist in developing standard care practices.

Teaching strategies of anatomical variations

The literature highlighted the need to increase the awareness of anatomical variations among medical students and clinical practitioners [9, 12, 41, 42]. This call came as a number of medical schools are moving away from dissection and shifting towards plastic anatomical models and digital software [12, 41]. Meanwhile, many medical schools teach anatomy to first-year medical students as typically described in atlas books, overlooking anatomical variations. Nevertheless, the literature shows a shortage in studies researching appropriate learning strategies for teaching the anatomical variations to junior medical students.

Previous studies emphasized that cadaver dissection is ideal for teaching anatomy [43, 44, 45]. Usually, students are assigned to dissect in groups of 6-8 students under the supervision of experts who are responsible for explaining concepts, such as anatomical variations, and their clinical application to students [43]. The long dissection hours, which may extend to 270 hours a year, give medical students the opportunity to explore and discuss with their peers many anatomical variations.

On the other hand, Kiss [44] shortlisted nine anatomical variations worth teaching to medical students, including variations in the circle of Willis, a missing dorsalis pedis artery, an internal carotid artery pseudoaneurysm inside the sphenoidal sinus, cervical ribs, a linguofacial trunk, arteria lusoria, variations of the coronary artery branches, variations of the liver anatomy, and variations of the cecum and appendix. However, students are commonly assigned to one cadaveric specimen, which decreases the chance to observe the listed variations. Thus, Sprunger [45] found that a cadaver reassignment system facilitates students’ appreciation of anatomical variations when they work on different specimens. Furthermore, a study suggested using innovative technology to create three-dimensional (3D) digital models of anatomical variations identified during routine dissection to teach and describe anatomical variations [46]. Thus, the 3D models will provide additional access for medical students to the identified variation along with the dissected specimen.

Other studies emphasized teaching anatomical variations in the clinical phase of medical school and postgraduate training programs. Besides learning the normal human morphology, medical students need to understand the concept of anatomical variations at the early stages of medical school and then strengthen this knowledge in the clinical years of the medical curriculum [47]. Similarly, Anscomb [48]explained the challenges to teaching and learning anatomical variation in postgraduate specialty programs. Postgraduate students should train using deep learning approaches to retain and apply the anatomical knowledge and related variations to achieve clinical best practice. Moreover, it has been found that surgical and radiological trainees learn more about anatomical variations from surgeons and radiologists than the specialty textbooks [10]. However, these variations were rarely assessed in their training curriculum. Thus, the educational leaders recommended more teaching on the anatomical variations in the early years of the resident surgical and radiological programs.

## Conclusions

In summary, anatomical variation is common within clinical practice and dissection sessions. Identifying these variations is essential for medical practitioners, as it may change the clinical practice routine. Approaches for identifying anatomical variations include cadaveric dissection, physical examination, preoperative imaging assessment, and surgical procedures. Observational studies, including cadaveric dissection or medical image evaluation and evidence-based anatomy, may increase the awareness of the anatomical variation. Dissection is a common learning tool in medical schools for teaching anatomy and relevant variations. However, the literature shows a gap in educational studies evaluating the learning and assessment of anatomical variations. Thus, the current study recommends creating a health informatics database to collect common anatomical variations encountered during clinical practice to enhance awareness of these variations and improve clinical practice outcomes. Finally, there is a need to conduct more research on the learning and assessment of anatomical variations in medical curricula.
